# Supplementary material for: Dietary choline in gonadectomized kittens improved food intake and body composition but not satiety, serum lipids, or energy expenditure
Source: PLoS One. 2022 Mar 17;17(3):e0264321. doi: 10.1371/journal.pone.0264321 (PMC8929592; doi:10.1371/journal.pone.0264321)
Supplement: Attachment — Submitted filename: Review of the article.docx [file pone.0264321.s001.docx]

**Review of the article No. PONE-D-21-36602**, entitled “Dietary choline in gonadectomized kittens improved food intake and body composition but not satiety, serum lipids, or energy expenditure”

The aim of the study was to assess the effect of dietary supplementation with choline in cats on selected parameters, which may be influenced by gonadectomy and subsequent possible obesity.

Overall, the authors have done a very good job describing their methods and analysing the data. The manuscript is well written, and results are easy to follow. It is worth to underlined, that the data obtained in the study are very important both from a dietary and physiological point of view. The discussion is very thorough and easy to read, and clearly presents the results and comparisons. The interpretations and conclusions are proved by the data.

In conclusion, excellent work!
